# Is Beauty in the Face of the Beholder?

**DOI:** 10.1371/journal.pone.0068395

**Published:** 2013-07-10

**Authors:** Bruno Laeng, Oddrun Vermeer, Unni Sulutvedt

**Affiliations:** 1 Department of Psychology, University of Oslo, Oslo, Norway; 2 Department of Psychology, University of Tromsø, Tromsø, Norway; Royal Holloway, University of London, United Kingdom

## Abstract

Opposing forces influence assortative mating so that one seeks a similar mate while at the same time avoiding inbreeding with close relatives. Thus, mate choice may be a balancing of phenotypic similarity and dissimilarity between partners. In the present study, we assessed the role of resemblance to Self’s facial traits in judgments of physical attractiveness. Participants chose the most attractive face image of their romantic partner among several variants, where the faces were morphed so as to include only 22% of another face. Participants distinctly preferred a “Self-based morph” (i.e., their partner’s face with a small amount of Self’s face blended into it) to other morphed images. The Self-based morph was also preferred to the morph of their partner’s face blended with the partner’s same-sex “prototype”, although the latter face was (“objectively”) judged more attractive by other individuals. When ranking morphs differing in level of amalgamation (i.e., 11% vs. 22% vs. 33%) of another face, the 22% was chosen consistently as the preferred morph and, in particular, when Self was blended in the partner’s face. A forced-choice signal-detection paradigm showed that the effect of self-resemblance operated at an unconscious level, since the same participants were unable to detect the presence of their own faces in the above morphs. We concluded that individuals, if given the opportunity, seek to promote “positive assortment” for Self’s phenotype, especially when the level of similarity approaches an optimal point that is similar to Self without causing a conscious acknowledgment of the similarity.

## Introduction

Current psychological research on human attractiveness has replaced the relativistic belief that “beauty is in the eye of the beholder” with a universalistic one. According to the latter account, our sense of facial beauty is not merely the result of arbitrary cultural values or personal idiosyncrasies but, to a greater extent, reflects features that are shared cross-culturally and appear early in development [1], [2], [3], [4]. Averageness, symmetry, and sexual dimorphisms of facial proportions (e.g., size and shape of the nose or eyebrows) are key features that serve the role of indicators for biologically relevant traits (i.e., health, reproductive potential, pro-social parenting behaviors). Although both averageness and symmetry would seem to be equally sought by males and females, sexual dimorphisms reflect each sex’s differing investments in reproduction.

However, the opposition between the relativistic and the universalistic perspectives may only be apparent, since one can posit the coexistence of an early, developmental, “imprinting” for physical traits of close con-specifics (typically, family members but also Self) as another universal mechanism that accounts for kin recognition as well as having an impact on mating preferences [5]. Indeed, face recognition mechanisms are heritable [6] and humans may be born with a schematic knowledge of the human face, which is then modified or filled out through exposure to human faces early in life. Thus, on one hand, a facial attribute like averageness would be based on a lifetime exposure to a large number of other con-specifics [7], so that one would expect that individuals within the same social group would tend to share a very similar (or seemingly “universal”) sense of what is the human average appearance. On the other hand, an imprinting mechanism, based on early experience, would lead to the opposite effect of establishing idiosyncratic “ideals” of beauty that may differ considerably between individuals. Thus, the coexistence of general learning mechanisms and mechanisms of kin recognition should shape ideals of facial (or bodily) aesthetics that are to a great deal consistent across many individuals but contain some elements that are unique to each individual. In particular, faces are known to play a special role in humans and there is a consensus that babies are already equipped with inborn information about the perceptual structure of faces and possess mechanisms that guide a preference for face-like patterns and thus facilitates the learning of facial identities at an early age [8]. For both sexes, general physical attractiveness is better predicted by ratings of facial attractiveness than by ratings of body images [9], [10].

A template-based hypothesis of facial attractiveness would be that a particular individual (or Self hereafter) will show attraction towards individuals showing moderate degrees of facial resemblance to Self [11], [12], [13] [14]. Several studies on actual couples have shown the presence of similar characteristics among spouses e.g., [11], [15], [16], [17], [18], [19], [20], [21], [22], [23], [24]. For example, when participants have been asked to sort pictures of unknown individuals of both sexes, photos of the actual partners were paired above chance [24]. In one study [25], jealousy responses for imaginary sexual infidelity scenarios based on stories were enhanced more if the photos were similar to self than if they were not.

Indeed, *positive assortative mating* (i.e., “like mate with like”; [26]) is the most common mating pattern found among animals [27] and clearly the term can also be applied to humans (sometimes referred to as “homogamy”; e.g. [28]). There are strong reasons to believe that the use of kin or ‘genetic’ similarity cues in sexual choice may be strategic in evolutionary terms. Laeng and colleagues [29] have previously described a narrow form of male narcissism for eye color (interpreted as a strategy for increasing paternal confidence and uncovering cuckoldry), where blue-eyed men are more attracted to women with the same eye-color. Most important, several studies indicate that a moderate degree of genetic similarity increases both reproductive success [30] and genetic compatibility [31]. For example, genealogical records of the whole population of Iceland (between 1800 and 1965) show a positive association between kinship and fertility [30]. Couples that were mildly related had the greatest reproductive success and the highest number of children who further reproduced. Specifically, there was a positive association between kinship and fertility (i.e., the number of children produced), with the greatest reproductive success observed for couples who are third or fourth cousins. The reproductive success of these Icelandic couples (i.e., the number of their children who reproduced) was described by a non-linear function where reproductive success starts off low for closely related couples (i.e., second cousins or closer), increases with relatedness, and peaks at third and fourth cousins, then decreases with relatedness and reaches its lowest values for distantly related couples (e.g., sixth cousins or beyond).

The study on Icelanders clearly indicates that 1) extreme genetic similarity between spouses can result in low reproductive success but that 2) moderate genetic similarity can be beneficial. Indeed, extreme assortative mating among humans should be limited by mechanisms of inbreeding avoidance [32], [33] as well as an opposing preference for some genetic diversity (e.g., for increased allelic diversity at the major histocompatibility complex; [34]) and a tendency to reduce outbreeding depression [12]. Therefore, humans may seek an optimal but delicate balance between outbreeding and inbreeding and we should expect sexual choice to be expressed towards face stimuli whose similarities to oneself are not too obvious (e.g., a face resembling too explicitly a sibling or a closely-related individual may trigger avoidance mechanisms of primary incest).

In the present study, we show facial images of attractive individuals of the opposite sex that have been previously manipulated (i.e., morphed) to contain different degrees of the facial shape of the participant and partner. One hypothesis is that the participant expressing the judgment or Self will be attracted to faces that show moderate degree of physical self-resemblance. Thus, we set up a series of experiments where participants were asked to choose the most attractive face image among several variants. Crucially, we expected that self-referential effects in physical attractiveness should be expressed towards face stimuli whose similarities to self are so subtle that they are not consciously apprehended [35].

## Experiment 1

Two people forming a couple and having a sexual relationship are likely to have chosen one another on the basis of a host of other criteria than physical self-resemblance [36] and any specific pairing of individuals may be the outcome of not only attraction but also of inability to obtain a more desirable mate, sheer opportunity, and chance encounters [37]. Thus, we would expect that, if Self plays a significant role for attractiveness, two lovers may actually prefer that their real-life partners resembled themselves to a greater degree than they actually do. The present experiments provided participants with the opportunity of making such a choice, although indirectly and without their knowledge.

Specifically, we asked partners in a stable romance/sexual relationship to rank the attractiveness of several versions of their partners’ faces. Using the face of one’s actual love partner would seem to have a clear advantage over using faces of strangers of the opposite sex. In fact, strangers’ faces could be judged unattractive by the participant on the basis of other, unpredictable, features or idiosyncratic associations (based on identity cues; e.g., “he reminds me of an unpleasant old schoolmate”) that could negatively dominate the aesthetic judgments (even at a subliminal level [38]) over and above the presence of self-referential features. In general, when people select mates, their traits come in a bundle [39] and the presence of one trait that is clearly below the threshold of attractiveness may make other attractive traits irrelevant. However, lovers, by definition, have already chosen each other and are, typically, sexually attracted to one another; therefore we would expect that adding Self’s features to their appearance could only enhance the perceived attractiveness. In order to reveal the presence of such a “narcissistic effect” in the present context, it would seem necessary to show that morphing Self into a partner’s face produces a better result than all other potentially attractive morphs and, in particular, than the morph of the partner’s face with its age cohort’s same-sex prototype. In addition, by comparing the morph of the partner’s face with same-sex and opposite-sex prototypes from the same age cohort, we can measure the degree to which androgyny reduces facial preferences, since morphing with the same-sex prototype will reduce androgyny while morphing with the opposite-sex prototypes will increase it. Thus, we generated an “androgynous morph” consisting of the partner’s face blended with the average of the two sex prototypes (i.e., the 50% morph of same-sex and opposite-sex prototype faces); such a morph contains a lower degree of androgyny compared to the other prototypes while at the same time it maximally enhances facial symmetry and averageness. Therefore, showing that the Self morph is preferred to any of these three prototype morphs should constitute rather strong evidence for the image of Self playing a significant role in face aesthetics.

In order to control for such a potential narcissistic effect, we asked the participants to evaluate the Self morphs generated for other couples. This control group should respond very differently to the Self morphs. In fact, we would predict that they would rank the prototype morphs (and in particular the opposite-sex morph) as more attractive than the morph based on the face of each model’s partner. In the control condition none of the images were morphed with the participant’s own face and the label ‘partner morph’ only indicated the same (highest ranked) pictures already used in the previous experiment. Each of the same twenty couples that participated in the first experiment was asked to judge the morphs previously generated for one of the other participating couples. In this case, the aesthetic judgments concerned paired individuals who were in neither a romantic nor a personal relationship with the judging couple.

As argued above, the morphing should be visually subtle in the graphic manipulations (i.e., “soft” morphs where the percentage contribution of other faces was 22%). We also limited morphing to the internal features of the face (i.e., the region containing and immediately surrounding the eyes, nose and mouth), while making sure that the outer contours of the faces were not affected. This procedure yields novel face images that strongly resemble the original face (since the hair, the outline of the face, and the overall head size remain unchanged) but yet contain in a subtle manner identity-relevant information from another face. Indeed, research on face perception has indicated that the internal or central portion of the face may contain the optimal features for identity [40] and be more important than the peripheral regions of the face for face identification (e.g., hair, ears and jaw line; [41]). Hence, we expected that such mild manipulations of the internal face information towards self-resemblance would be sufficient to trigger narcissistic responses without the observer being necessarily aware of “seeing” Self (cf. [35]).

### Methods

#### Participants

All participants were Norwegian and residents of the town of Tromsø, Norway. Psychological research in Norway is subject to ethical review by the regional medical research board only if the research involves patients, children or animals and involves use of drugs, genetic samples or invasive techniques. Since none of these conditions applied to the present study, the academic institution demanded only that the project comply with Declaration of Helsinki guidelines and that informed consent be obtained from the participants. We obtained written informed consent from all participants. All information was handled and stored anonymously, while respecting privacy and secrecy, and participants were free to withdraw from the project. In addition, participants gave their written informed consent, as outlined in the PLOS consent form, to publication of their photographs.

Twenty young heterosexual couples (*N* = 40) participated in the experiment. Each pair of lovers had been together for a minimum of two years. The participants’ mean age was 28 years (*SD* = 5). Each participant was shown 7 different images of their partner and asked to rank the images based on their attractiveness or sexual appeal.

#### Procedure

**Figure 1:**
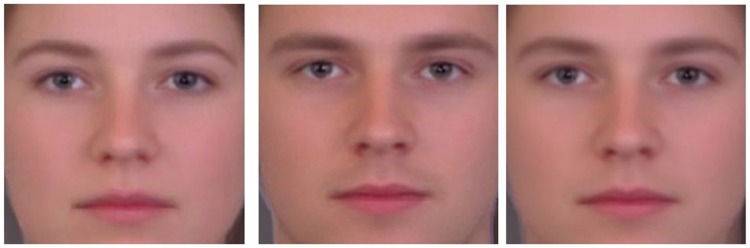
The Prototypes. Examples of the female prototype (left), male prototype (middle), and androgyne prototype (right).

All couples were invited to visit the lab and a frontal, close-up, photo of each individual was taken with the same background and the same digital camera in the same lighting conditions and distance from the model’s face. The original photo were then edited in Adobe Photoshop® and morphed images were generated by use of Morpheus software®. One morph consisted in a 22% blend of the participant’s face in that of the partner, so as to create the ‘Self’ morph; two other morphs were obtained by 22% blends with the “prototypical” female face or the “prototypical” male face (each of these being morphs of 30 females or 30 males, respectively, drawn from the same age and ethnic group of the participants; see Figure 1).

**Figure 2:**
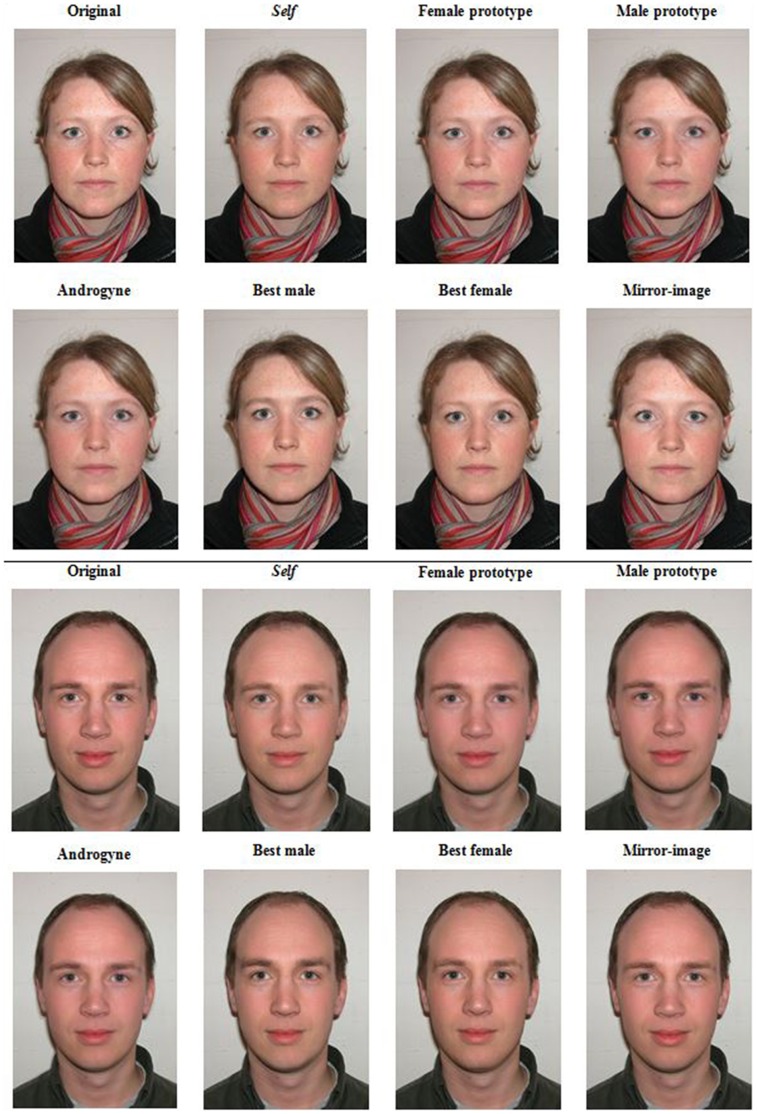
The Morphs. Examples of the original image and of the seven 22% morphs of one participating couple (female: top two rows; male: bottom two rows). Nota Bene: The ‘Self’ image is a morph obtained blending the ‘original’ face of the participant ranking the images.

Another morph image was generated by first averaging the two sex prototypes (i.e., blending 50% of the same-sex prototype with 50% opposite-sex prototype so as to obtain a combination of the 30 female and 30 male “parent” faces) and then using the obtained ‘androgynous’ image to contribute 22% of the final morph with the partner’s face, here labeled as the “androgyne morph”. Two more morphs were created using two of the participants’ faces, of the same and opposite sex, that had been rated as the most attractive of the sample by external judges (N = 20; 10 females), these constituting the “best female morph” and the “best male morph”. Finally, a “mirror morph” was created for each participant’s face by blending (50%) the original face with a mirrored, horizontally flipped, version of the same picture. The latter manipulation was included since a well-known side effect of the morphing process is that the faces become more symmetrical and that the texture of the skin appears smoother than that of its component pictures; thus, this “mirror morph” maintains strong likeness to the original face, but it is equally smoother in appearance and may be more symmetric than the other morphs. We limited morphing to the internal features of the face (i.e., the region containing and immediately surrounding the eyes, nose and mouth) by selecting out with use of Adobe Photoshop the central, oval, region of the face and then pasting it onto the original photograph, smoothing the edges, so as to obtain an image where all of the external features of the face (e.g., hair and jaw line) and clothing remained identical in each variant (see Figure 2).

In the control condition each participant couple was randomly assigned to one set of pictures of another couple, each consisting of the same 7 pictures previously evaluated by partners. Images were ranked from most (1) to least attractive, by observing ad-lib high-quality color paper prints of all of the morph images.

### Results

Given that we obtained ranks for the different morphs (i.e., ordinal data), all results were analyzed using the Friedman’s Rank Test, which is the non-parametric alternative to the one-way repeated-measures analysis of variance. Pairwise comparisons were carried out with the non-parametric Paired Sign Test. Two separate Friedman’s Rank Tests were performed by splitting participants by sex (see Table 1).

**Table 1:** Mean ranks of the 7 morphs as evaluated within the couple.

*Male Participants*	*Mean rank*
*Self morph*	1.70
*Female prototype morph*	2.63
*Androgyne morph*	2.80
*Male prototype morph*	3.70
*Best female morph*	4.33
*Mirror morph*	6.20
*Best male morph*	6.65
***Female Participants***	***Mean rank***
*Self morph*	1.45
*Androgyne morph*	2.63
*Female prototype morph*	3.15
*Male prototype morph*	3. 63
*Best female morph*	4.58
*Best male morph*	6.03
*Mirror morph*	6.55

The analysis of male participants’ ranks revealed a systematic preference, *χ^2^* = 88.6, *df* = 6, *p*<.0001. The Self morph was ranked first and the female prototype morph was second (p = .0026); therefore the Self morph was significantly superior to all the other morphs (.0026<*p*<.0001) as assessed with Paired Sign tests, which are non-parametric analyses that allow comparisons between ordinal data sets. Interestingly, the female prototype and androgyne morphs did not differ significantly from each other (*p* = .263). However, the androgyne morph was significantly preferred to the male prototype morph (*p* = .0414) and the best female prototype morph was preferred to the mirror morph (*p*<.0001).

The analysis of female participants’ ranks also revealed a systematic preference, *χ^2^* = 86.5, *df* = 6, *p*<.0001. The Self morph was ranked first, followed by the female prototype (p = .012); thus the Self morph was again significantly superior to all the other morphs (.012<*p*<.0001). The three prototype morphs did not differ significantly from each other (.115<*p*<.824) whereas the best female morph was significantly different from the mirror morph (*p* = .003).

As expected, the control condition showed that the male participants ranked the female prototype morph first and, most importantly, the partner morph was ranked last (see Table 2), *χ^2^* = 57.15, *df* = 6, *p*<.0001. The three prototypes were ranked on top and did not significantly differ from one another,.79<p<.99. The partner morph differed significantly from all three prototypes (p<.0001). The control female participants expressed similar preferences and, again, they ranked the female prototype morph as first and the partner morph last, *χ^2^* = 39.71, *df* = 6, *p*<.0001. Again, the three prototypes were ranked on top and did not significantly differ from one another,.18<p<.79. The partner morph differed significantly from all three prototypes (.001<p<.0001).

**Table 2:** Mean ranks of the 7 morphs as evaluated by another couple.

*Male Participants*	Mean rank
*Female prototype morph*	2.11
*Androgyne morph*	2.21
*Male prototype morph*	2.25
*Best female morph*	4.29
*Mirror morph*	5.29
*Best male morph*	5.79
*Partner morph*	6.07
***Female Participants***	**Mean rank**
*Female prototype morph*	2.14
*Androgyne morph*	2.43
*Male prototype morph*	3.00
*Best male morph*	4.50
*Best female morph*	4.61
*Mirror morph*	5.36
*Partner morph*	5.96

The ‘Partner morph’ images consisted of the same images labeled as ‘Self morph’ in Table 1.

In order to compare ranks between the couples and the controls, we performed separate simple regression analyses of the ranks obtained by the different groups of participants. Based on our hypotheses, we do not expect males and females to differ from one another in their preferences for the various morphs, instead we would expect their rankings to be highly similar or correlated. A simple regression of ranks of male participants and female participants in the couples’ group showed a highly significant positive relationship, R = .997, Y = 0.14+0.97, F(1,6) = 105.7, p<.0001, confirming that the preference for the different morphs were nearly identical for both sexes. In contrast, when each of these groups’ ranks were correlated to the controls’ ranks, we found that there was no significant relationship between ranks of couples’ male participants and controls’ male participants, F(1,6) = 1.02, p = .36, as well as between ranks of couples’ female participants and controls’ female participants, F(1,6) = .31, p = .60.

### Discussion

Lovers clearly prefer their partners' faces to resemble their own over having their partners' faces look “more attractive” or more similar to the average face of their sex. Thus, these findings based on attractiveness of face manipulation of partners support the existence of a robust but context-dependent mating strategy that promotes positive assortment for facial resemblance based on Self’s phenotype. In addition, the three prototype morphs did not differ significantly in preference from one another. Interestingly, the androgyne morph did not either significantly lower or raise a prototype’s rank in attractiveness for participants of either sex, thus suggesting that the elements of androgyny contained in the present soft (22%) morphs did not constitute a significant confound.

The results from the control condition differed considerably from those of the experimental condition, and these results confirm that the partner morphs were not previously chosen simply because they accidentally happened to comply with some shared standard of beauty. If the Self morph images were better stimuli than the others, also participants unrelated to the target faces would show agreement with the previously observed Self morph’s rankings. Interestingly, studies of actual matches in couples have also shown that lovers of similar attractiveness are drawn to one another as romantic partners [42], [43], [44] or that they would prefer a partner similar to themselves [45]; although when given a chance to choose a hypothetical partner (in either the laboratory or speed-dating situations), participants may often prefer partners that are more attractive than themselves [46], [47].

## Experiment 2

We hypothesized that two opposing forces that a) seek assortative mating and b) avoid inbreeding with close relatives, should yield mate choices that are a balance of phenotypic similarity vs. dissimilarity between partners. Therefore, in a follow-up experiment, we used face morphs to assess the existence of a preference on attractiveness judgments of different levels of morphing, namely 11%, 22% and 33% of a participant’s face or the same-sex prototype blended within the face of the partner. In other words, we expect that avoidance mechanisms of primary incest would forbid a too strong resemblance of the participant to the target face and, on the other hand, too weak a resemblance may fail to trigger the ‘like seeks like’ strategy. A previous study by Fraley and Marks [35] had participants provide attractiveness ratings for 4 levels of Self morphing (22%, 32%, 39%, and 45%) onto faces of opposite-sex strangers; although they found that the 22% morphs were preferred to the original faces (0% morphing), all morphs were rated equally attractive.

In the present study, we again asked participants to rank morphs, thus forcing choices between alternatives. We expected that the 22% would be preferred to a stronger contribution of Self’s face, i.e., a 33% contribution. However, we also expected that a weaker contribution, i.e. a percentage of 11%, would result in a loss of preference for the Self-based morph. In the previous experiment, the 22% morph was successful in revealing a preference for images that included Self’s face and therefore, in the present experiment, we tested two additional levels of morphing at the same distance (in morphing percentages) from the previous level of morphing but in opposite directions.

As a comparison, we also included 11%, 22% and 33% morphs with the same-sex (of the partner) prototype. Differently from the Self morph, we expected that the stronger “dose” (i.e., 33%) of the prototype would be preferred to other weaker ones (i.e., 11% and 22%).

Note that in the present experiment, we assume that the presence of Self was invisible at a conscious level in all blends, since they all contained a low percentage (i.e., a maximum of 33%) of self’s internal features of the face onto the target face (leaving untouched the outer shape and hair in the original image).

### Methods

#### Participants

The participants (*N* = 20) were 10 of the couples that had participated in the previous experiment and had already signed an informed consent form.

#### Procedure

We used the same morphing procedures used in Experiment 1, except that three different levels of morphing were used to obtain the 11%, 22% and 33% morphs with *Self* and the prototype faces. The resulting images maintained a sharp resemblance to the “target” face with no ambiguity about identity. Again, during the experiment, all morphs were displayed simultaneously in color and on paper and each participant was asked to rank them in order of attractiveness. It was pointed out that “attractive” should also be interpreted as “sexy”.

### Results

Descriptive statistics were first calculated for each participant, obtaining mean ranks for each morph. A preliminary analysis showed no differences in ranking between males and females; hence a single analysis based on all participants was used (see Table 3). There was a systematic preference for different morphs, *χ^2^* = 60.2, *df* = 6, *p*<.0001. The Self 22% morph was ranked first, followed by the prototype 33% morph. Importantly, the Self 11% and 33% morph were at the bottom of the ranking.

**Table 3:** Experiment 2: Mean ranks of the 7 morphs.

	*Mean rank*
*Self 22% morph*	1.47
*Prototype 33% morph*	2.16
*Prototype 22% morph*	4.11
*Mirror morph*	4.66
*Self 11% morph*	4.90
*Prototype 11% morph*	5.13
*Self 33% morph*	5.58

### Discussion

Attractiveness judgments of morphs of the observer’s face with faces of opposite-sex partners were clearly modulated by similarity to the observer. Different doses of resemblance to ‘self’ caused changes in attractiveness judgments of the morphs, resulting in the Self 22% morph being preferred to all of the others. The Self morphs that had lower (11%) or higher (33%) doses of similarity to *Self* were the least preferred versions of the partner’s face. These findings are consistent with our assumption that a 22% Self morph approximates the “sweet spot” balancing the inbreeding-outbreeding opposing tendencies. In addition, the present findings suggest that the prototype face, which should trigger no inbreeding avoidance, is tolerated at higher level of amalgamation (i.e., 33% was most preferred) than that allowed for the phenotype-based traits.

A previous study by Fraley and Marks [35] had also hypothesized the existence of an optimal point of self-resemblance and tested the effects on sexual attractiveness of 4 levels of morphing (22%, 32%, 39%, and 45%) as well as no morphing (0%) onto faces of opposite-sex strangers. Although Fraley and Marks found that the 22% morphs were preferred to the original, non-manipulated faces, it appeared that all of the morphs were found equally attractive. In contrast, we found a decrease in preference for a stronger morph (33%) stronger than 22% as well as for a morph with a weaker contribution of Self (i.e. 11%). Based on our findings, we can extrapolate that the loss of preference may have been greater for even stronger morphs. This seems reasonable in the light of a study by Turk et al. [48] on a split-brain patient that used systematic 10% step increases of morphing of the patient’s face into that of another, highly familiar, individual (e.g., Michael Gazzaniga’s face). The patient’s left hemisphere showed an inability to explicitly recognize self in morphs where his face contribution was lower than 30% and the same drop in performance occurred at a an earlier point for the right hemisphere (60% of self). Thus, previous studies using 50% blends have typically failed to find Self-similar enhancements of preference but they appear to have been successful when using lower percentages (e.g. with 25% blends [49]).

Fraley and Marks’s study failed to reveal the non-monotonic changes in attractiveness along the variable of similarity to Self that they had actually predicted on the basis of Bateson’s model of optimal outbreeding [5]. One key methodological difference between their study and the present one is that each morphing percentage of Self was applied to different opposite-sex strangers instead of the face of a same individual as in the present study. Moreover, we controlled the attractiveness levels of the test faces, since we used the faces of partners (i.e., individuals deemed attractive by each participant). Also, we opted for rankings instead of ratings as the dependent variable, since ratings may fail to reveal subtle differences between hedonic estimates that can be better teased apart by forcing the observers to make a choice. Therefore, using ratings may have obscured other effects than a generic preference for morphed images, perhaps due to their enhanced averageness and smoothness of features compared to the unmanipulated face (a possibility that we had directly controlled by including a “mirror” morph as well as prototype morphs). Nevertheless, the present findings do support Fraley and Marks’s conclusions and offer a straightforward account for previous failures to revealing effects of Self similarity (i.e., by using too strong “doses” of Self).

## Experiment 3

Evolutionary accounts do not require that individuals are aware of either the reasons for their preferences or what elements of a stimulus trigger their decisions and feelings [50], [51]. Indeed, some of the most relevant preferences from an evolutionary viewpoint may occur unconsciously and when made aware they may be subjected to revision or ‘editing’ and possibly lead to less spontaneous responses and a change towards more socially accepted choices [35]. Several psychological studies have revealed that stimuli processed unconsciously can activate a broad variety of processes [52], as shown for example by research on subliminal perception of emotional expression [53], [54], [55] as well as of attractiveness [56], [57]. In addition, “mere exposure” at the subliminal level [58] can produce significant changes in the affective responses to the unconsciously processed stimuli. Interestingly, sexually attractive stimuli can also powerfully attract attention even when they are completely “invisible” (i.e., non-reportable or undetected) to the observers. Jiang and colleagues [59] have shown by use of the interocular suppression paradigm that such suppressed erotic pictures, albeit invisible, can attract the observers’ spatial attention. An unconscious attentional bias towards one stimulus among several others may also be sufficient to form, through a feedback loop, an aesthetic preference for the attended stimulus over the others [60].

Platek and colleagues [61], [62] have shown that males react differentially towards children’s faces that resemble them (e.g., when the stimulus child was a 25% morph of the observer), although the participants are unaware of the effects of resemblance on their choices. Other neuroimaging studies have revealed strong brain activity to subliminal presentations of the names of beloved ones compared to subliminal presentations of the names of friends [63]. Remarkably, these neuroimaging studies also imply the existence of a face processing network for discrimination of non-kin from kin [64].

Most relevantly, Fraley and Marks [35] showed that subliminally presented faces of a participant’s parent (i.e., a 17 ms presentation of a “kin prime” followed by a 17 ms mask) increased the attractiveness ratings of a subsequent stranger’s face. Remarkably, the verbal suggestion that a participant’s face had been morphed into the test faces (though no manipulation had actually been made) was sufficient to significantly lower their attractiveness. Possibly, the conscious knowledge that the faces being rated may be genetically related may have been sufficient in triggering a culture-based mechanism of incest avoidance.

However, according to some accounts, awareness may not be an all-or-none phenomenon but it can also be conceived as varying gradually [65] so that one could suppose that in the present morphed stimuli the resemblance to Self might be consciously seen, albeit weakly, and only remain at the “fringe” [66]. That is, when confronted with weak signals, observers may fail to report a target simply because they have low confidence in the detection and this may bias participants to appear unaware. One recommendation for ruling out the above possibility is to use “objective criteria” of awareness, by having participants perform forced-choice detection tasks [67], [68]. In contrast, asking participants at the debriefing stage whether they noticed something unusual during the task or if they became aware of the graphic manipulation constitutes an example of a “subjective” test of consciousness, since participants are requested to provide a verbal report. The “objectivity” of forced-choice detection would derive from the requirement of making a choice even in conditions in which differences can only slightly be discriminated and by subsequently analyzing, through ‘signal detection theory’ procedures [69], both the sensitivity to the difference in stimuli and the degree of neutrality, conservativeness, or liberality in making a specific choice.

Thus, if our participants could weakly detect self-resemblance but were not confident enough about it to report it openly, then the use of a forced-choice detection task in detecting the presence of Self in morphs should reveal it. Specifically, participants saw one 22% morph face of their partner in each trial and decided whether the image contained their own face or not. The following morphs appeared with equal probabilities: A Self morph, an ‘Other’ morph (i.e., a morph with the face of another participant of the same sex, matched by age and complexion), and a target face (or partner) ‘Mirror’ morph. Participants were informed of the equal probabilities of each type of stimulus and requested to always make a choice about the presence of Self or its absence and to indicate to what degree they were confident of each decision. The Other morphs were included in order to control for the possibility that participants could “guess” the difference between ‘mirror’ morph and Self morph on the basis of low-level differences (e.g., overall symmetry or slight differences in luminance of specific face regions). Finally, all responses were analyzed according to signal-detection theory [69], by obtaining a *d’* measure of sensitivity for each individual participant.

### Methods

#### Participants

The participants (*N* = 40) were the same 20 heterosexual couples that participated in the previous experiment and had already signed an informed consent form.

#### Stimuli

Each participant’s partner face was morphed with a 22% contribution of the participant’s face (Self morph), or with another participant of the same sex, matched by age and complexion (the ‘Other’ morph). In addition, we selected the horizontally flipped image of the partner’s face (the ‘Mirror’ morph).

#### Procedure

Each participant was informed that they would see a series of faces, one at the time, and they had to decide whether each face contained elements of the participant’s face. At the beginning of the experiment, each participant was familiarized with the morphing technique by interactively viewing on the computer screen the morphing layouts (in Morpheus Photo Morpher^©^) for all three types of morphed images. By moving the cursor on the morphed image display, each participant could appreciate how it is possible to generate images that contain contribution of two pair of faces in variable amounts of visibility. Participants were then informed that, during the task, one third of the face stimuli would contain their own face, albeit in a small amount, and that the rest of the pictures would contain the face of a stranger, in the same small amount, or no other image than the face of their partner. Participants were also told that the morphs may be difficult to distinguish from each other but that their task was to always make a choice about a) whether the face looked like themselves or not and b) after each choice they would also have to indicate how confident they were about their decision on a scale from 1 (very low confidence) to 6 (very high confidence). There were a total of 120 trials in the whole test; that is, 40 trials per condition. Stimulus presentations were controlled by SuperLab^©^ software, which also stored each key press. Participants sat at a comfortable distance of 72 cm from the screen and saw each image centered on a 17 inches computer screen in full-screen mode for 1 second, after which the screen turned blank. The participant made a key press by selecting one of two digit keys on the keyboard labeled ‘yes’ (i.e. Self) or ‘no’ (i.e. ‘Other).

### Results

We calculated descriptive statistics for each participant by obtaining rates of hits, misses, false alarms, and correct rejections for stimuli were the target signal was present (i.e., Self) and those where the target signal was absent (‘Other’ or ‘Mirror’ morphs). Then we obtained each individual’s Sensitivity measure (*d’*) together with its Criterion score (*C*) for each type of noise target (i.e., either happy or neutral noise targets were considered separately); *d'* assesses how well two things can be distinguished and *d'* ranges from 0 (no discrimination) to infinity (perfect discrimination). A *d’* of 4 or more indicates nearly perfect performance; whereas when *C* = 0 then an observer's criterion is 'neutral', showing no decision bias towards one or other response type (yes or no).

We computed 95% confidence limits, according to the formula of Macmillan and Creelman’s [69], for the average *d’* scores of ‘Self’ versus ‘Other’ (mean *d’ = *.34; C.I._0.95_ = .57) and ‘Self’ vs. ‘Mirror’ (mean *d’ = *.47; C.I._0.95_ = .58) and found that neither mean departed significantly from a *d’ = *0 (i.e., no sensitivity). We also computed 95% confidence limits for the average *C* scores of ‘Self’ versus ‘Other’ (mean *C* = .36; C.I._0.95_ = .39) and ‘Self’ vs. ‘Mirror’ (mean *C* = .36; C.I._0.95_ = .38) and confirmed that neither mean departed significantly from *C = *0 (i.e., neutrality in the observers’ criterion).

“Confidence” scores in the forced choices were analyzed with ANOVA tests. Confidence was high, ranging from 3.6 (for ‘hits’ with Self morphs) to 4.8 (for ‘correct rejections’ with ‘Other’ morphs). There was no difference in average confidence ratings for each of the morphs (‘Self’ = 4.2; ‘Other’ = 4.1; ‘Mirror’ = 4.3), F(2,38) = 1.4, p = .69.

### Discussion

We used an “objective test” of consciousness [67] and found no evidence that Self morphs could be distinguished from other morphs, since our participants could not detect better than chance that an image of the partner had been blended with Self from either an image of the partner blended with ‘Other’ (i.e., a stranger) or from the original (‘Mirror’) face.

We reasoned that humans may prefer an optimal balance between outbreeding and inbreeding and that an “incest taboo” avoids extreme inbreeding at a conscious level [35]. By showing that our participants were unaware of the presence of Self’s face in the present morph stimuli strengthens the conclusion that self-referential effects can be revealed at levels of similarity between an observer and the opposite-sex face that remain unconscious.

A previous study on self-similarity [70] did not use morphs but an interactive face transformation technique where participants were allowed to manipulate the appearance of an opposite-sex face along a continuum from a self-similar face, through an average face to a face with opposite facial features. It was found that attractiveness ratings increased with similarity, but such a relationship declined (and reached asymptote) when faces became too similar to the observer. Such results are also consistent with the existence of an optimal outbreeding point. Interestingly, in the same study, there was a trend for attractiveness ratings of self-similar faces to be higher than the ratings of the same face images by other observers. Given that in the above study the self-resembling manipulation became explicit during the experiment, we surmise that participants might have made choices that were more conservative or “socially acceptable” (thus closer to those of other raters) than they could have been if the nature of the manipulation had been unknown to them.

## General Discussion

A glance at a face can be enough to provoke trust, aversion, or sexual attraction. Physical resemblances to Self and/or childhood attachment figures are placed at the core of these choices by evolutionary accounts. Indeed, much of the process responsible for attractiveness among individuals of the opposite sex seems to occur outside of awareness. The present study shows that, at least at an unconscious level, individuals of both sexes do love their partners as they are but also like themselves to such an extent that they prefer a photographic version of their partner’s face that contains a small amount of their own facial traits. Specifically, a self-referential morph was preferred over the morph of the partner’s face with the latter’s same-sex prototype. Crucially, in the experiments, other individuals (i.e., member of the other participating couples) consistently ranked as most attractive the morphs of the partner’s face with the latter’s same-sex prototype whereas the morph of the same face with the partner’s face was ranked by these control judges as the least attractive. Thus, when given the opportunity, romantic partners may prefer that their partners’ faces resemble their own over having their partners' faces “objectively” look more attractive. In this respect, the present findings bring some support to the “matching hypothesis” originally proposed by some social psychologists [42], [71], [72] [73] that men and women of similar attractiveness are drawn to one another as romantic partners as a reflection of direct biases rather than simply as an indirect (side) effect of each individual’s ability to attract and compete with other (available) individuals within the “biological market” [74], [75].

One proposed mechanism behind active assortative mating is that the “template” of the sought-after physical traits is based on that of human kin detection, which operates by computing estimates of genetic relatedness between self and other on the basis of two ancestral cues: a) the perinatal association with the individual’s biological mother, and b) duration of sibling co-residence. This kin recognition process is also based on facial phenotype matching [76], especially for the recognition of older siblings [77]. The ability to match facial phenotypes would allow detecting kin status in other, non-familiar, individuals [78]. Developmental studies on human babies have shown that early experience at 6 to 9 months of age in individuating faces can critically shape the perceptual mechanisms for later recognition and discrimination of faces [79], [80].

Note that such an “imprinting” process does not exclude that self-inspection with mirrors would also influence the formation of the kin template. In fact, the face we are probably most familiar with, and already at a very early age, is our own [81]. Thus, a “proximate” mechanism for facial imprinting may be based on the “mere exposure” phenomenon [21], so that highly familiar faces tend to be regarded as more likeable and attractive. However, an imprinting process goes beyond mere exposure effects, since it would seem to imply a sensitive period as well as other experiential factors [82], [83]. Importantly, humans learn to recognize themselves in a mirror in the first years of life, a process that has been given central importance in psychological developmental theories [84]. The recognition of one’s image in the mirror (e.g., the “Rouge test”) is considered as evidence of being conscious of owning a body, a face, and being a “Self” separate from others [85], [86]. Other animals that demonstrate highly developed cognitive and empathic abilities (i.e., apes, elephants and dolphins) also show signs of self-recognition in mirrors [86], [87], [88]. In sum, we assume that humans “imprint” to Self’s face (via reflections on shiny surfaces, like mirrors, as well as photos and films) and that this process contributes to shaping an individual’s standard of facial ‘beauty’.

An evolutionary “ultimate” mechanism for a phenotypic similarity bias between partners could be based on inclusive fitness [89], [90], [91], [92], [93]. Increasing the coefficient of parent-offspring as well as grand-offspring’s genetic relatedness [5], [94] can result in increased gene duplication without an increase in reproductive investment and with a reduced cost of altruism [27], [95]. For example, the benefit of helping a full sibling would increase because of assortative mating between the parents. Moreover, assortative mating for personality or cognitive traits may make cooperation between nonrelatives (i.e., “reciprocal altruism”) more effective. As mentioned, there is also evidence for a relation between genetic relatedness and increased fertility in humans [30]. According to Thiessen and Gregg [27], individuals will attempt to “capture” as many homologous genes as possible by assorting with mates who are similar, while attempting to avoid mating among consanguineous individuals [32], [96], [97], [98].

Moreover, biologists have pointed out that a selection against extreme outbreeding could be adaptive because it prevents co-adapted gene complexes from breaking up [31], [99], [100], [101], [102]. A co-adapted gene complex is a group of genetic traits which have high fitness when they occur together, but which without each other have low fitness. Since active mating choices must be based on external visible cues, it is possible that an effective preventive strategy could then be that of seeking mates that are similar to the Self phenotype.

Additional benefits from positive assortment in humans may accrue on the basis of reducing costs that affect rearing of the offspring; for example, psychological and physical similarities between spouses can increase marital satisfaction, levels of love, commitment, and the likelihood that two parents will stay together [103], [104], cooperate effectively in the support of their children [105], and ultimately, increase their evolutionary fitness [106]. Positive assortment on the basis of facial similarity would also seem to increase parents-to-offspring similarity, as facial appearance has a strong genetic base [107], which might have the effect of increasing paternal confidence [108]. Hence, resemblance in facial features may be sought by males to reduce the costs of rearing someone else’s offspring, as well as being used by females as a strategy for increasing their partners’ confidence and secure support to the family [29], [109].

To conclude, the maxim that “beauty is in the eye of the beholder” is not incompatible with the process of assortative mating or with the idea that “principles” of human mate choice are universal. If these “constraints” may be universal, the results can be highly contextual, since the cues of assortative mating are based on learning [50]. Several studies suggest that the early exposure to prevalent bodily traits of peers or kin can potently shape sexual preferences, that will be shown later in adult life, towards those very traits (e.g., the prevalent gender of kin or schoolmates can modulate preference for masculinity or femininity [110], and a different skin color of childhood nurses can enhance later the sexual attraction to other, but specific, ethnicities [111], p. 278). Charlotte Brontë’s *Jane Eyre* (1847) best expressed this: “Most true is it that beauty is in the eye of the gazer.” Several of the prominently preferred facial traits may show little variation among adult humans, cultures, and ethnicities [112], since these traits are important for one’s reproductive success or the survival success of the offspring, regardless of specific environmental and social contexts. However, some of the traits that are considered as most desirable of potential mates may have also evolved to be based on similarity to traits possessed by the beholder. It is in this sense that Brontë’s maxim is not at all inconsistent with a universalistic, evolutionary, view of beauty.
